# Supplementary material for: In silico hippocampal modeling for multi-target pharmacotherapy in schizophrenia
Source: NPJ Schizophr. 2020 Sep 21;6:25. doi: 10.1038/s41537-020-00109-0 (PMC7506542; doi:10.1038/s41537-020-00109-0)
Supplement: Supplementary file 1 — Supplementary Information [file 41537_2020_109_MOESM1_ESM.pdf]

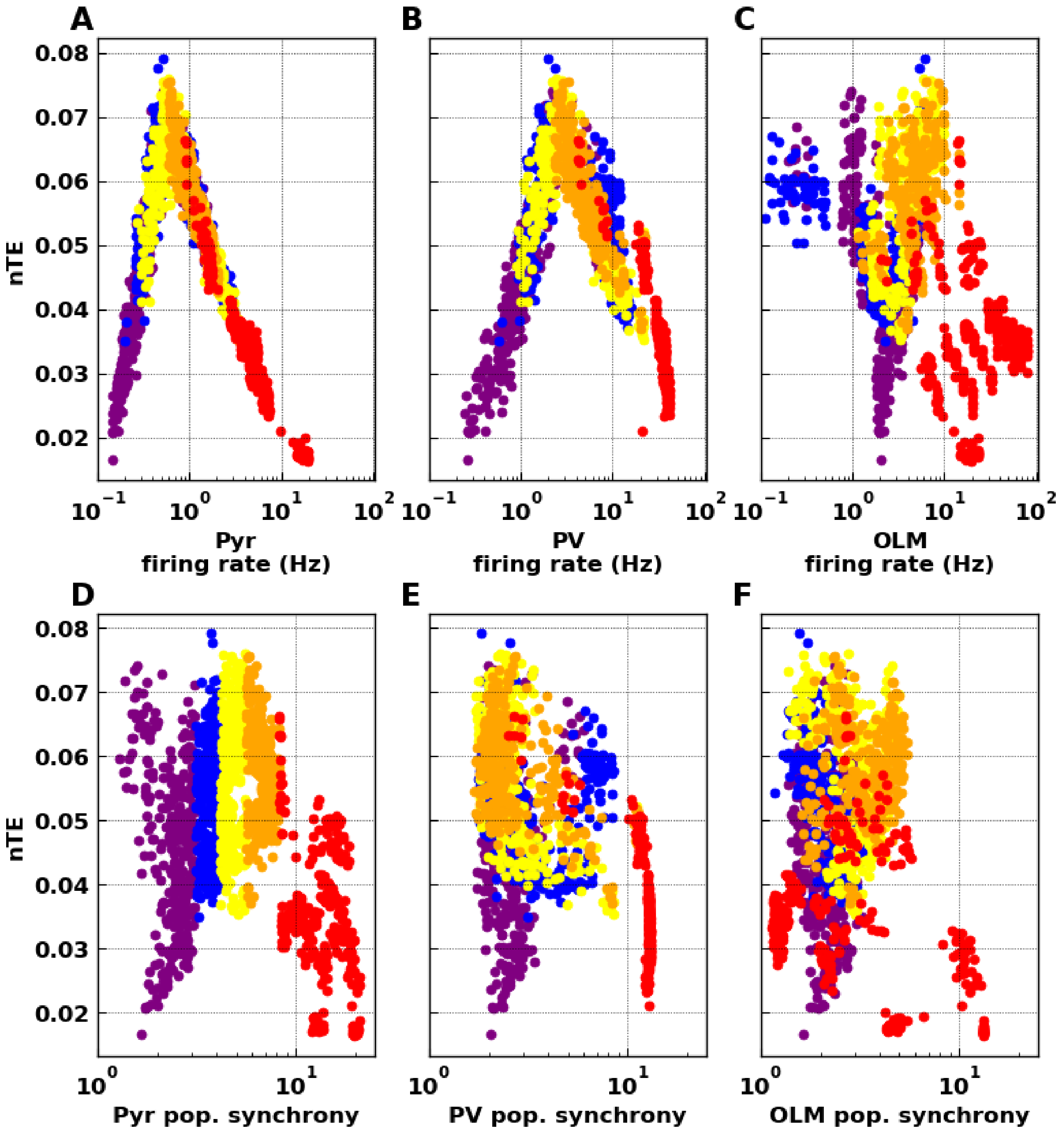

Suppl. Fig. 1: Pyramidal and basket cell population firing rates and synchrony were associated with information flow changes.
Variability of population firing measured with population synchrony. Points in the panels, representing individual simulations, are color-coded according to quintiles measured for pyramidal population synchrony. For pyramidal and PV populations, quintiles show a general consistency across these 4 measures. There was no clear relationship between OLM interneuronal firing pattern and information flow (panels C and F).
